# Theoretical Studies of the Spin-Dependent Electronic Transport Properties in Ethynyl-Terminated Ferrocene Molecular Junctions

**DOI:** 10.3390/mi9030095

**Published:** 2018-02-26

**Authors:** Shundong Yuan, Shiyan Wang, Zhaoyang Kong, Zhijie Xu, Long Yang, Diansheng Wang, Qidan Ling, Yudou Wang

**Affiliations:** 1 College of Science, China University of Petroleum, Qingdao 266580, China; wangshiyan@upc.edu.cn (S.W.); kongzhy@outlook.com (Z.K.); xuzj@upc.edu.cn (Z.X.); dshw@upc.edu.cn (D.W.); 2 Research Institute of Experiment and Detection of PetroChina Xinjiang Oilfield Company, Karamay 834000, China; zy-yl@petrochina.com.cn; 3 College of Materials Science and Engineering, Fujian Key Laboratory of Polymer Materials, Fujian Normal University, Fuzhou 350007, China; lingqd@fjnu.edu.cn

**Keywords:** ferrocene, density functional theory, nonequilibrium Green’s function, electron-transport property, spin polarization

## Abstract

The spin-dependent electron transport in the ferrocene-based molecular junctions, in which the molecules are 1,3-substituted and 1,3′-substituted ethynyl ferrocenes, respectively, is studied by the theoretical simulation with nonequilibrium Green’s function and density functional theory. The calculated results suggest that the substitution position of the terminal ethynyl groups has a great effect on the spin-dependent current-voltage properties and the spin filtering efficiency of the molecular junctions. At the lower bias, high spin filtering efficiency is found in 1,3′-substituted ethynyl ferrocene junction, which suggests that the spin filtering efficiency is also dependent on the bias voltage. The different spin-dependent transport properties for the two molecular junctions originate from their different evolutions of spin-up and spin-down energy levels.

## 1. Introduction

The molecular device is thought to be the substitute for the silicon-based microelectronic device for application in future computers. Over the recent twenty years, molecular electronics has been investigated widely by researchers all around the world. Experimental and theoretical explorations of electronic transport properties in molecular devices have made great progress [1,2,3,4].

Relative to the conventional electronic device, the spintronic device has many advantages, such as nonvolatility, smaller size, and faster data processing speed [5]. Spin in an organic semiconductor may show some attractive transport properties, such as a long spin relaxation time. Therefore, as a newly-emerged cross discipline of molecular electronics, organic spintronics, and molecular magnetism, etc., molecular spintronics has also received a lot of attention by researchers [6,7,8,9,10,11,12,13,14,15,16,17]. Due to the unique spin transport properties in molecules, molecular spin devices are expected to be applied to future electric circuits. In spite of the great progress in the technology and method, there are still many uncertainties in the experimental preparation and measurement of molecular spin devices because of their ultra-small size. Some transport mechanisms in molecular spin devices also still need to be clarified. Therefore, it is necessary to use a theoretical simulation to investigate the electron transport for the specific models of molecular spin devices so that the experimental results and the working mechanism can be understood. Furthermore, the molecular spintronic devices with particular function may be designed and fabricated with the help of theoretical simulation. Currently, the most commonly employed theoretical method is nonequilibrium Green’s function (NEGF) combined with density functional theory (DFT) [18,19].

Metallocene is a complex with a transition metal cation sandwiched between cyclopentadienyl (Cp) rings, and it has unpaired spin components and good chemical stability. Considering it as a possible candidate for molecular devices, the molecular junctions based on metallocene were investigated extensively through experiments and theoretical simulations [20,21,22,23,24,25,26,27,28,29,30,31]. Some spin-dependent transport properties, such as spin filtering and spin switching, were found.

**Figure 1:**
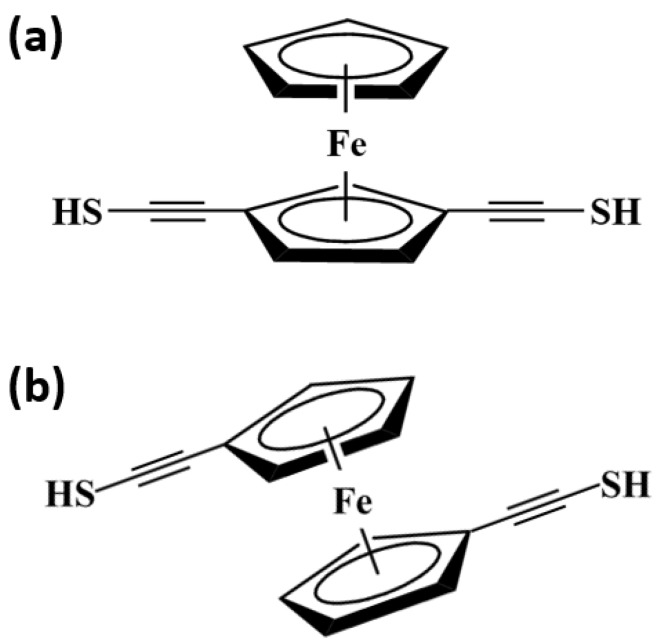
Chemical structures of (**a**) 1,3-substituted and (**b**) 1,3′-substituted thiolated ethynylferrocene molecules.

Ferrocene (FeCp_2_) is the most common metallocene. The two Cp rings can be in the staggered or eclipsed conformation and rotate with low resistance about the Cp–Fe–Cp axis [25,30,32]. Nemnes et al. investigated the electron transport of the FeCp_2_-based molecular junctions, in which the two FeCp_2_ conformations and the different electrode–electrode distances were considered [30]. Using first-principles methods, Morari et al. studied the electron-transport properties in a 1,1′-ferrocene dicarboxylic acid molecular junction. In their works, the different bondings of end-groups to the electrodes were mainly considered [26]. In organic molecules, alkynyl is usually introduced to modulate the molecular rigidity, linearity, and electronic structure. Various oligoferrocenylacetylenes have been synthesized through linking ferrocene with alkynyls [33,34]. The work of Zhang et al. shows that the transport properties will be altered when alkynyl is introduced into polyferrocenylene [27]. For a ferrocene monomer with two ethynyl fragments, many molecular structures may be present, just as shown in Figure 1. Our previous work showed that the spin-dependent electron transport may be influenced by the molecular configuration for ethynyl-linked biferrocene molecule [35]. For the FeCp_2_-based nanowires, the spin transport properties can be affected by the number of FeCp_2_ units, i.e., the molecular length, as some works have suggested [36,37]. The smaller molecule may be made into the smaller molecular junction with a more stable configuration, which is more meaningful for future application in nano-level circuits. Moreover, the electronic structures of the smaller molecules are usually easier to be influenced by chemical modification relative to the bigger molecules, thus the electronic transport properties are easier to be modified to achieve the required device function [38]. Therefore, for the ethynyl-terminated ferrocene molecules with different configurations, spin-dependent electron transport is worthwhile to be studied.

In this work, the spin-dependent electron transport properties of ferrocene-based molecules linked to Au electrodes by the thiolated terminal ethynyl groups, as shown in Figure 1, are investigated using the theoretical NEGF−DFT method. Although there may be some differences for the transport properties between the eclipsed and staggered conformations of the ferrocene molecule [30], the different molecular structures caused by the substitution position of the terminal ethynyl groups are considered emphatically in this work. Therefore, only the eclipsed FeCp_2_ conformation is adopted for the sake of brevity. The two ethynyl groups on ferrocene monomer are 1,3-substituted and 1,3′-substituted, respectively. The effect of substitution position of ethynyl on the spin-dependent electron transport is discussed.

## 2. Computational Models and Methods

**Figure 2:**
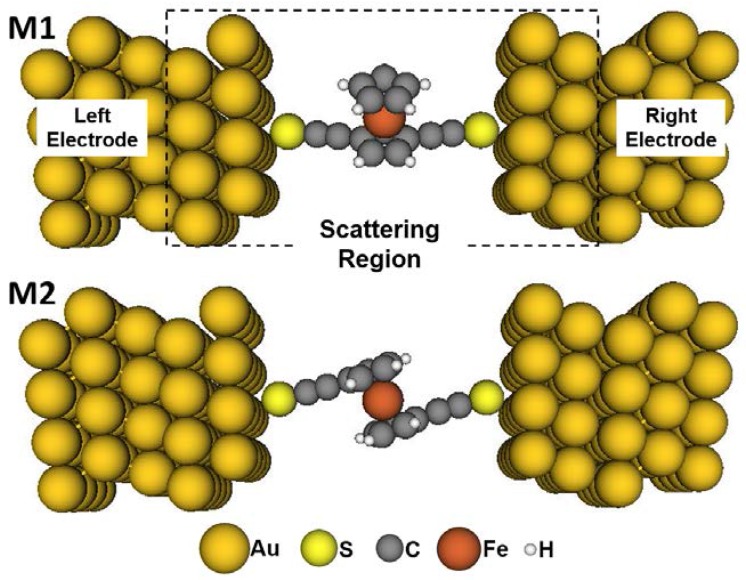
Models M1 and M2 of the ethynyl ferrocene molecular junctions. Au, S, C, Fe, and H atoms are represented by the golden, yellow, gray, brown, and white balls, respectively.

Figure 2 illustrates the models of molecular junctions, and they are labelled M1 and M2, respectively. When the thiol group is linked to gold electrode, a gold-sulfur covalent bonding is formed to fasten the molecule. The molecular geometry in Figure 1 is optimized before building the junction model. The optimization is performed through Gaussian03 program [39] at the DFT/B3LYP [40,41] level of theory with 6-31G(d,p) basis set for S, C, and H atoms, and LANL2DZ basis set for Fe atom.

The next procedure is the model construction of the molecular junction. As shown in Figure 2, the optimized molecule is placed between the two gold electrodes which are modeled by two Au (111)–(4 × 4) surfaces with periodic boundary conditions. The molecule and the central four gold layers comprise the scattering region. The sulfur atoms on the molecular terminal are placed on the hollow sites of the gold surfaces. The gold–sulfur distance is 2.30 Å, which is the same as our previous computational models [35,42]. For the molecular junctions similar to the present ones in which the molecule is attached to the gold electrode via the terminal thiolated ethynyl, the change in the molecular configuration may be ignored. Therefore, the Au/molecule/Au structures in this work are not further optimized, as with the treatment in our previous work [35]. Subsequently, the spin-dependent transport calculations for models M1 and M2 can be carried out. For the main calculation parameters, a double-*ζ* with polarization (DZP) basis set is used for all atoms of the molecule, and a single-*ζ* with polarization (SZP) basis set is used for Au atoms. The Perdew–Zunger local density approximation (LDA.PZ) is employed for the exchange-correlation potential [43,44]. The convergence criterion of the grid integration is 1 × 10^−5^. The mesh cutoff is set as 250 Ry. The *k*-point sampling in the self-consistent calculation is 4 × 4 × 500 for the Brillouin zone integration. All the above model construction and transport computation are accomplished through VNL/ATK package (Version 2008.10, QuantumWise, Copenhagen, Denmark) [45], and its theoretical basis is NEGF combined with DFT.

At an applied bias *V*_b_, the spin-dependent current *I*_σ_ (σ represents spin up and spin down states) flowing through the molecule is calculated by the Landauer–Büttiker expression [46]:

(1) $$I_{\sigma }(V_{b})=\frac{e}{h}\int [f_{L}(E-\mu_{L})-f_{R}(E-\mu_{R})]T_{\sigma }(E,\;V_{b})dE$$

where *h* is the Planck’s constant, $f_{L/R}$ is the Fermi function of the left/right metal electrode, $\mu_{L/R}$ is the chemical potential of the left/right electrode: $\mu_{L}(V_{b})=E_{F}-eV_{b}/2$, $\mu_{R}(V_{b})\;=\;E_{F}+eV_{b}/2$. $E_{F}$ is the Fermi energy of the electrode. $T_{\sigma }(E,\;V_{b})$ is the spin-dependent transmission function for electron with energy *E* and is obtained by

(2) $$T_{\sigma }(E,\;V_{b})=T_{r}[\Gamma_{L\sigma }G_{\sigma }^{R}\Gamma_{R\sigma }G_{\sigma }^{A}]$$

where $G_{\sigma }^{R(A)}$ is the retarded (advanced) Green’s function of the central scattering region with spin index σ, $\Gamma_{L(R)\sigma }$ is the spin-dependent coupling matrix between the scattering region and the left (right) electrode.

## 3. Results and Discussion

**Figure 3:**
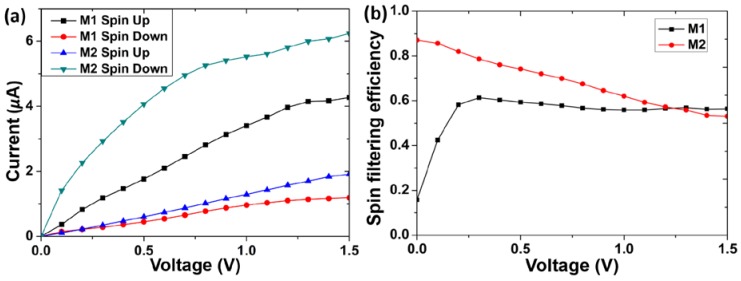

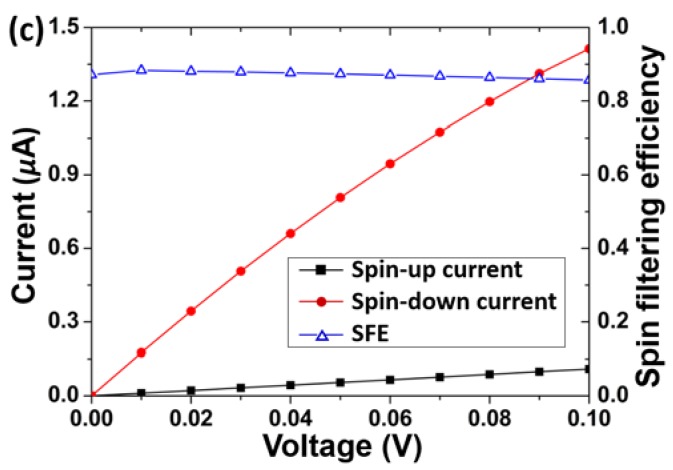
(**a**) Spin-polarized *I*-*V* curves of models M1 and M2; (**b**) Spin filtering efficiency (SFE) curves of models M1 and M2; (**c**) Spin-polarized *I*-*V* curves and SFE curve of model M2 at the lower bias voltages.

Figure 3a shows the spin-polarized current-voltage curves of models M1 and M2 from 0 V to 1.5 V. For model M1, the spin-up current values increase throughout, and the *I-V* curve displays an approximate linear evolution trend except for the part of the higher voltages. The spin-down current values for model M1 increase slowly with the increasing bias voltages, and they are less obvious than the corresponding spin-up current values. For model M2, when the bias is applied, the spin-up current values increase slowly, and its curve also displays an approximate linear trend. On the contrary, the spin-down current values increase rapidly from 0 V to 0.7 V, and then the trend slows down. For each model, there is an obvious deviation between the spin-up and spin-down curves. Therefore, the electric currents flowing through the two molecules show the spin-polarized characteristics.

To evaluate the spin-polarized characteristics in two molecules, the spin filtering efficiency (SFE) at a bias voltage is defined as [16]

(3) $$\mathrm{SFE}=\left|\frac{I_{\mathrm{spin}-\mathrm{up}}-I_{\mathrm{spin}-\mathrm{down}}}{I_{\mathrm{spin}-\mathrm{up}}+I_{\mathrm{spin}-\mathrm{down}}}\right|\times 100\%$$

where *I*_spin-up_ and *I*_spin-down_ represent the current values of the spin-up and spin-down states, respectively. The SFE at zero bias can be obtained by the equation

(4) $$\mathrm{SFE}=\left|\frac{T_{\mathrm{spin}-\mathrm{up}}-T_{\mathrm{spin}-\mathrm{down}}}{T_{\mathrm{spin}-\mathrm{up}}+T_{\mathrm{spin}-\mathrm{down}}}\right|\times 100\%$$

where *T*_spin-up_ and *T*_spin-down_ represent the transmission coefficients of the spin-up and spin-down states, respectively. The larger SFE value means a better spin filtering effect. If the SFE value of the device approaches 100%, it can serve as a perfect spin filter. In Figure 3a, the current values show the interesting characteristics of *I*_spin-up_ > *I*_spin-down_ in model M1 but *I*_spin-up_ < *I*_spin-down_ in model M2. This may be attributed to the different energy level structures between M1 and M2 as discussed in the following text. Additionally, it should be noted that the current values of model M1 show an opposite characteristic compared to our results in the previous work [35], in which the current values of model M1 composed of biferrocene molecule show *I*_spin-up_ < *I*_spin-down_, and the corresponding energy level structures should also be responsible for this. The calculated SFE values from 0 V to 1.5 V of models M1 and M2 are shown in Figure 3b. It can be seen that the two curves show different evolution tendencies. The SFE values of model M1 keep constant approximately (about 60%) above 0.2 V. It means that model M1 has a stable and good spin filtering effect at the higher bias. In model M2, the SFE values exceed 80% at the lower bias and decrease obviously with the increasing bias voltages before 1.4 V. Therefore, our result indicates that the spin polarization of the thiolated ethynylferrocene molecule in this work is related to the substitution positions of the terminal ethynyl groups. In addition, the spin polarization is also modulated by the applied bias. In our previous work [35], the SFE values of the two models employing with biferrocene molecule show the upward trend roughly from 0 V to 1.5 V. This is different from the results in the current work. The difference comes from the different evolution trends of *I*_spin-up_ and *I*_spin-down_. Our results suggest that the SFE can be affected by the number of ferrocene unit. In the lower bias, the ferrocene-basd junctions have more excellent SFE than biferrocene-based junctions.

In model M2, the SFE values are 87% at 0 V and 85.7% at 0.1 V. It is supposed that the SFE values below 0.1 V are always high. Thus, the current values of model M2 in the bias range of [0, 0.1] V with a step of 0.01 V are calculated, and the results are presented in Figure 3c. The spin-up current values increase very slowly with the rising biases; on the contrary, the spin-down current values increase rapidly. Thus, the current values between spin-up and spin-down states show a large difference. The corresponding SFE values of model M2 is also presented in Figure 3c. As in our supposition, all of the SFE values are close to 90% (the highest is 88.3%). The high spin filtering efficiency is very important for designing spintronic devices. According to the spin polarization characteristics of models M1 and M2, it suggests that the ethynyl-terminated ferrocene molecules may be used for designing a stable and high-performance spin filtering device.

The transmission spectrum can be employed to analyze the above-current voltage characteristics. According to Equation (1), the transmission coefficients within the so-called bias window [$\mu_{L}(V_{b})$, $\mu_{R}(V_{b})$] determine the electric current. The Fermi level of the electrode is not fixed and it will shift when a bias is applied. If *E*_F_ value at zero bias is set to 0, the bias window is [−*V*_b_/2, +*V*_b_/2].

**Figure 4:**
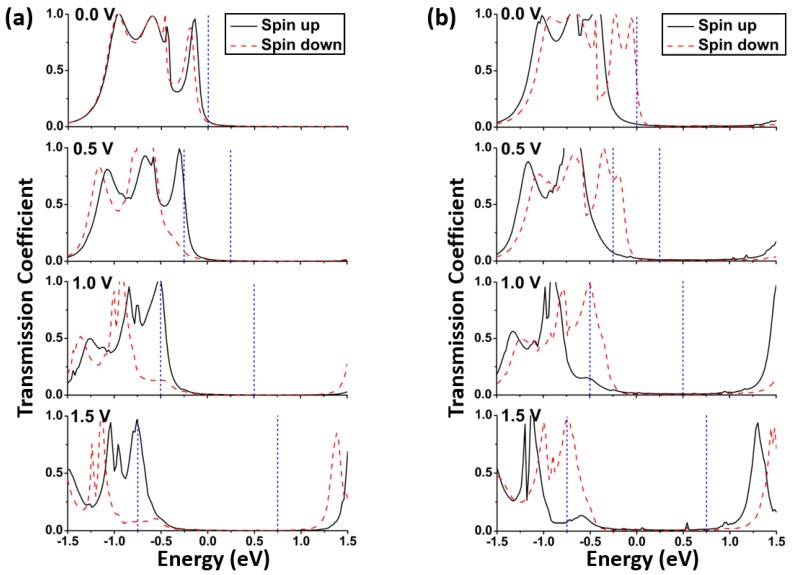
Spin-up and spin-down transmission spectra at the different biases for two models: (**a**) M1; (**b**) M2. The region confined by the two blue vertical dashed lines is the bias window.

The transmission coefficients of spin-up and spin-down states in the energy region of [−1.5, 1.5] eV in model M1 at 0 V, 0.5 V, 1.0 V, and 1.5 V are presented in Figure 4a, respectively. A larger integral area under the transmission spectrum can bring a larger electric current. At 0 V bias, there is little difference for the transmission coefficients between the spin-up and spin-down states at the energy of *E*_F_. At the higher biases, i.e., 0.5 V, 1.0 V, and 1.5 V, the spin-up transmission spectra in the bias window are significantly higher than the corresponding spin-down ones. Therefore, the spin-up integral areas are greater than the spin-down ones. It results in the relation of *I*_spin-up_ > *I*_spin-down_ in model M1. Similarly, for model M2, the spin-up and spin-down transmission spectra are shown in Figure 4b. Contrary to model M1, the spin-down transmission spectra of model M2 in the bias window are prominently higher than the spin-up ones. Thus, the current values show *I*_spin-up_ < *I*_spin-down_ in model M2.

**Figure 5:**
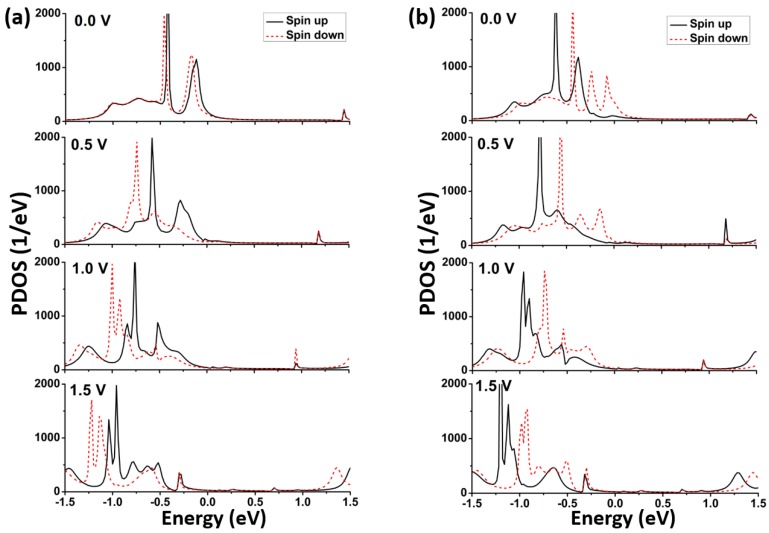
Spin-up and spin-down projected density of states (PDOS) spectra at the different biases for two models: (**a**) M1; (**b**) M2.

The formation of peaks on the transmission spectrum is connected with the molecular orbitals. The contribution of molecular orbitals on the transmission can be analyzed by the projected density of states (PDOS). The PDOS is the density of states which is projected onto some specific atoms of the model. The spin-up and spin-down PDOS curves in two models at the biases of 0.0 V, 0.5 V, 1.0 V, and 1.5 V are plotted in Figure 5. Here, the density of states is only projected onto the central molecule and the electrode atoms are not included. Comparing Figure 5 with Figure 4, it can be seen that the PDOS peaks well match the transmission ones; that is, the transmission may be prominent at the energy level where the PDOS peak appears. Meanwhile, the spin-up PDOS spectra in the bias window in model M1 are higher than the spin-down ones, and the case of model M2 is on the contrary. Additionally, it can be seen that the PDOS peaks shift toward the lower energy range when the bias is increased. These characteristics of PDOS spectra are coincident with those of transmission spectra.

**Figure 6:**
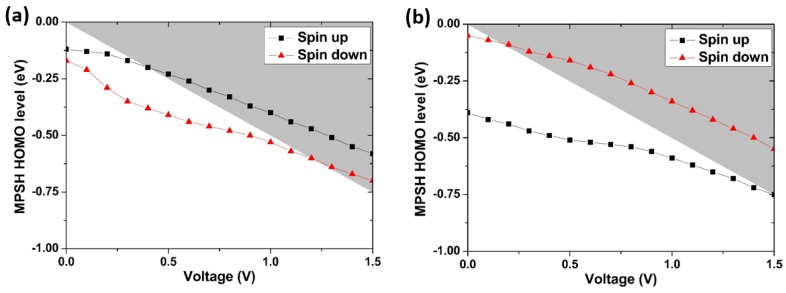
Highest occupied molecular orbital (HOMO) energy levels at the different biases for two models: (**a**) M1; (**b**) M2. The gray shaded area is the bias window.

To further understand the spin-dependent transmission characteristics, the molecular projected self-consistent Hamiltonian (MPSH) of the models is investigated. In our work, the Hamiltonian is projected onto the molecule in the middle of the model. For model M1 at zero bias voltage, the highest occupied molecular orbital (HOMO) levels are about −0.12 eV for spin-up state and −0.17 eV for spin-down state, and the lowest unoccupied molecular orbital (LUMO) levels are about 2.03 eV for spin-up state and 2.01 eV for spin-down state. These MPSH energy levels are in accordance with the positions of transmission peaks and PDOS peaks at zero bias voltage. Thus, the transmission peak in the bias window in model M1 originates from the HOMO level. Since *E*_F_ has been set to 0 eV, therefore, *E*_F_ is closer to the HOMO than to the LUMO. It indicates that the HOMO level is the decisive contributor of electric conductance in model M1. A similar situation is also found in model M2. The evolution of HOMO levels of models M1 and M2 is shown in Figure 6. For model M1, the spin-up HOMO levels are always located in the bias window from 0.4 V to 1.5 V. However, the spin-down HOMO levels do not enter the bias window until the bias is increased to 1.2 V. Consequently, the spin-up transmission is more prominent than the corresponding spin-down transmission. The evolution of spin-up and spin-down HOMO levels in model M2 is contrary to that in model M1, and it is also in accordance with the characteristics of transmission spectra of model M2.

**Figure 7:**
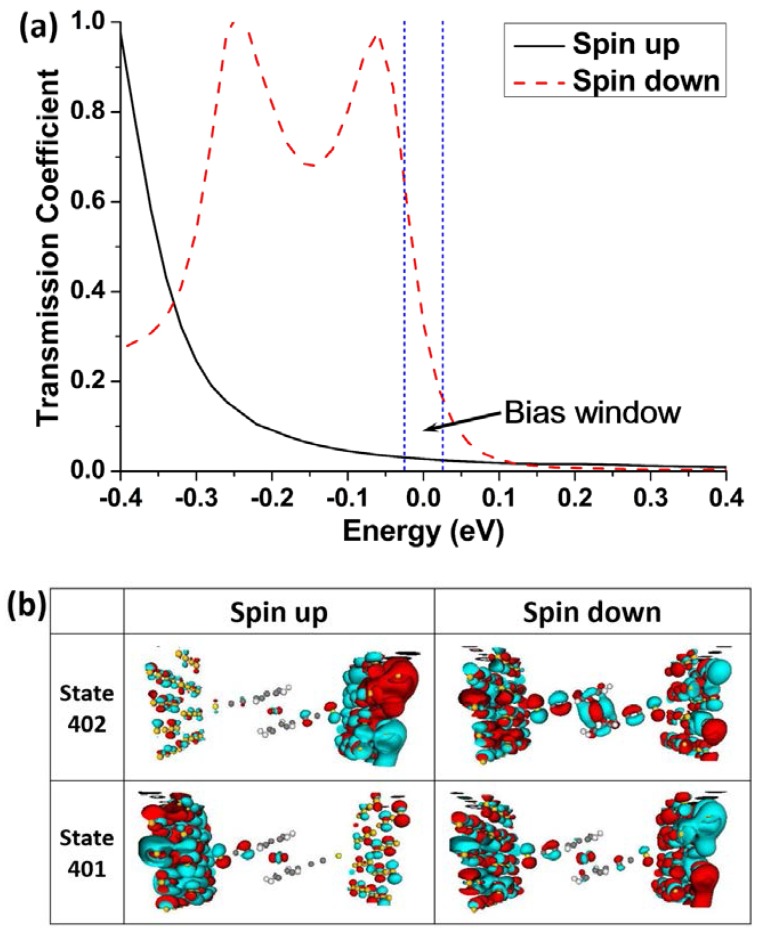
(**a**) Spin-up and spin-down transmission spectra at 0.05 V for model M2; (**b**) Spatial distribution of the molecular projected self-consistent Hamiltonian (MPSH) states 401 and 402 at 0.05 V for model M2. The isovalue is 0.03.

In order to analyze the high SFE value at the lower bias in model M2, the spin-up and spin-down transmission spectra at 0.05 V are compared, as shown in Figure 7a. In the bias window, the spin-down integral area is significantly greater than the spin-up one. The feature is consistent with the difference of currents at 0.05 V. In addition, the spatial distribution of MPSH state can be used to identify the molecular orbitals intuitively which contribute to the electron transport. When the Hamiltonian is projected onto the whole scattering region, i.e., including the molecule and 64 Au atoms, the number of energy levels increases. The MPSH states contribute to the electron transport only when they are delocalized on the whole scattering region [47,48]. After obtaining the MPSH level, the spatial distributions of electron states for the atoms in the scattering region at 0.05 V are calculated. In the energy range of the bias window at 0.05 V, most of the MPSH states are localized on the electrodes (see Figure S1 in Supplementary Materials), and they do not contribute to the electron transport. The spin-down MPSH state 402 shows well delocalization, and state 401 also shows a little delocalization, as shown in Figure 7b. The delocalized states give channels for electron transmission and thus bring a large current. On the contrary, the corresponding spin-up MPSH states 402 and 401 are localized. The same analysis method can also be applied to model M1. The spatial distributions of MPSH states 390-406 which are located in the energy range of the bias window at 0.5 V for M1 are calculated (see Figure S2 in Supplementary Materials). They confirm the transport characteristic of *I*_spin-up_ > *I*_spin-down_.

The total current *I*_tot_ under a bias voltage is given by *I*_tot_ = *I*_spin-up_ + *I*_spin-down_. According to this relation, it can be found from Figure 3a that the total current values in model M2 are larger than those in model M1. Therefore, the substitution position of the ethynyl on FeCp_2_ is important for the molecular conductance. Comparing to the Fe atom in model M1, the one in model M2 may make a more contribution to the electron tunneling from one electrode to the other electrode passing through the molecule, and the conductivity is enhanced accordingly. Therefore, this suggests that the participation of Fe atom should be a more intrinsic reason for the molecular conductance. The characteristic is similar to that in our previous work [35].

## 4. Conclusions

In this work, the spin-dependent transport properties of the molecular junctions based on ferrocene with terminal ethynyl groups are investigated by theoretical simulation with the NEGF−DFT method. For the two models M1 and M2, which are constructed from 1,3-substituted and 1,3′-substituted ethynyl ferrocenes, respectively, our calculated results show that their currents have spin-polarized characteristics. For model M1, the spin-up currents are greater than the corresponding spin-down ones, but the opposite is true for model M2. The SFE values of model M1 keep constant approximately in a broad bias range, and those of model M2 are high at the lower bias. The substitution position of the ethynyl group has a great effect on the spin-dependent transport properties and SFE. The results suggest that ethynyl ferrocene may be treated as a candidate for designing a stable and high-performance spin filtering device.

## Supplementary Materials

The following are available online at http://www.mdpi.com/2072-666X/9/3/95/s1, Figure S1: Spatial distribution of spin-up and spin-down MPSH states 391-402 at 0.05 V for model M2, Figure S2: Spatial distribution of spin-up and spin-down MPSH states 390-406 at 0.5 V for model M1.

## References

[1] Chen J., Reed M.A., Rawlett A.M., Tour J.M. (1999). Large on-off ratios and negative differential resistance in a molecular electronic device. Science.

[2] Taylor J., Brandbyge M., Stokbro K. (2002). Theory of rectification in tour wires: The role of electrode coupling. Phys. Rev. Lett..

[3] Tao N.J. (2006). Electron transport in molecular junctions. Nat. Nanotechnol..

[4] Aradhya S.V., Venkataraman L. (2013). Single-molecule junctions beyond electronic transport. Nat. Nanotechnol..

[5] Wolf S.A., Awschalom D.D., Buhrman R.A., Daughton J.M., von Molnar S., Roukes M.L., Chtchelkanova A.Y., Treger D.M. (2001). Spintronics: A spin-based electronics vision for the future. Science.

[6] Cornia A., Seneor P. (2017). SPINTRONICS: The molecular way. Nat. Mater..

[7] Sanvito S. (2011). Molecular spintronics. Chem. Soc. Rev..

[8] Shiraishi M., Ikoma T. (2011). Molecular spintronics. Physica E.

[9] Rocha A.R., Garcia-Suarez V.M., Bailey S.W., Lambert C.J., Ferrer J., Sanvito S. (2005). Towards molecular spintronics. Nat. Mater..

[10] Mas-Torrent M., Crivillers N., Mugnaini V., Ratera I., Rovira C., Veciana J. (2009). Organic radicals on surfaces: Towards molecular spintronics. J. Mater. Chem..

[11] Emberly E.G., Kirczenow G. (2002). Molecular spintronics: Spin-dependent electron transport in molecular wires. Chem. Phys..

[12] Camarero J., Coronado E. (2009). Molecular vs. inorganic spintronics: The role of molecular materials and single molecules. J. Mater. Chem..

[13] Coronado E., Yamashita M. (2016). Molecular spintronics: The role of coordination chemistry. Dalton T..

[14] Shultz D.A., Kirk M.L. (2014). Molecular spintronics: A web themed issue. Chem. Commun..

[15] Wu Q., Zhao P., Liu D., Li S., Chen G. (2014). Rectifying, giant magnetoresistance, spin-filtering, newgative differential resistance, and switching effects in single-molecule magnet Mn(dmit)_2_-based molecular device with graphene nanoribbon electrodes. Org. Electron..

[16] Deng X.Q., Zhang Z.H., Tang G.P., Fan Z.Q., Sun L., Li C.X. (2016). Modulation of the spin transport properties of the iron-phthalocyanine molecular junction by carbon chains with different connection sites. Org. Electron..

[17] Deng Y., Chen S., Zeng Y., Zhou W., Chen K. (2017). Large spin rectifying and high-efficiency spin-filtering in superior molecular junction. Org. Electron..

[18] Taylor J., Guo H., Wang J. (2001). Ab initio modeling of quantum transport properties of molecular electronic devices. Phys. Rev. B.

[19] Brandbyge M., Mozos J.-L., Ordejón P., Taylor J., Stokbro K. (2002). Density-functional method for nonequilibrium electron transport. Phys. Rev. B.

[20] Engtrakul C., Sita L.R. (2008). Ferrocene-based nanoelectronics: Regioselective syntheses and electrochemical characterization of α-monothiol and α,ω-dithiol, phenylethynyl-conjugated, 2,5-diethynylpyridyl- and pyridinium-linked diferrocene frameworks having an end-to-end distance of ~4 nm. Organometallics.

[21] Liu R., Ke S.H., Baranger H.U., Yang W.T. (2005). Organometallic spintronics: Dicobaltocene switch. Nano Lett..

[22] Liu R., Ke S., Baranger H.U., Yang W. (2006). Negative differential resistance and hysteresis through an organometallic molecule from molecular-level crossing. J. Am. Chem. Soc..

[23] Liu R., Ke S.H., Yang W.T., Baranger H.U. (2006). Organometallic molecular rectification. J. Chem. Phys..

[24] Liu R., Ke S., Yang W., Baranger H.U. (2007). Cobaltocene as a spin filter. J. Chem. Phys..

[25] Zhou L., Yang S., Ng M., Sullivan M.B., Tan V.B.C., Shen L. (2008). One-dimensional iron-cyclopentadienyl sandwich molecular wire with half metallic, negative differential resistance and high-spin filter efficiency properties. J. Am. Chem. Soc..

[26] Morari C., Rungger I., Rocha A.R., Sanvito S., Melinte S., Rignanese G. (2009). Electronic transport properties of 1,1′-ferrocene dicarboxylic acid linked to Al(111) electrodes. ACS Nano.

[27] Zhang G., Qin Y., Zhang H., Shang Y., Sun M., Liu B., Li Z. (2010). Electronic structure-transport property relationships of polyferrocenylene, polyferrocenylacetylene, and polyferrocenylsilane. J. Phys. Chem. C..

[28] Matsuura Y. (2013). Current rectification in nickelocenylferrocene sandwiched between two gold electrodes. J. Chem. Phys..

[29] Matsuura Y. (2013). Spin transport in bimetallocene. J. Appl. Phys..

[30] Nemnes G.A., Nicolaev A. (2014). Transport in ferrocene single molecules for terahertz applications. Phys. Chem. Chem. Phys..

[31] Abufager P.N., Robles R., Lorente N. (2015). FeCoCp3 molecular magnets as spin filters. J. Phys. Chem. C..

[32] Coriani S., Haaland A., Helgaker T., Jorgensen P. (2006). The equilibrium structure of ferrocene. ChemPhysChem.

[33] Butler I.R., Boyes A.L., Kelly G., Quayle S.C., Herzig T., Szewczyk J. (1999). Precursors towards poly-1,1′-ferrocenylacetylene: A simple synthesis of hetero-disubstituted ferrocenylethynes. Inorg. Chem. Commun..

[34] Plenio H., Hermann J., Sehring A. (2000). Optically and redox-active ferroceneacetylene polymers and oligomers. Chem. Eur. J..

[35] Yuan S., Wang S., Wang Y., Ling Q. (2017). Effect of molecular structure on spin-dependent electron transport in biferrocene-based molecular junctions: A first-principles study. J. Comput. Electron..

[36] Shen X., Yi Z., Shen Z., Zhao X., Wu J., Hou S., Sanvito S. (2009). The spin filter effect of iron-cyclopentadienyl multidecker clusters: The role of the electrode band structure and the coupling strength. Nanotechnology.

[37] Matsuura Y. (2018). Tunnel magnetoresistance of ferrocene molecules. Chem. Phys. Lett..

[38] Yuan S., Dai C., Weng J., Mei Q., Ling Q., Wang L., Huang W. (2011). Theoretical studies of electron transport in thiophene dimer: Effects of substituent group and heteroatom. J. Phys. Chem. A.

[39] Frisch M.J., Trucks G.W., Schlegel H.B., Scuseria G.E., Robb M.A., Cheeseman J.R., Montgomery J.A., Vreven T., Kudin K.N., Burant J.C. (2004). Gaussian 03.

[40] Becke A.D. (1993). A new mixing of Hartree–Fock and local density-functional theories. J. Chem. Phys..

[41] Lee C., Yang W., Parr R.G. (1988). Development of the Colle-Salvetti correlation-energy formula into a functional of the electron density. Phys. Rev. B.

[42] Yuan S., Wang S., Wang Y., Xu Z., Ling Q. (2016). Theoretical study of electron transport properties of bimolecular junctions: Effect of molecular arrangement and species. Comp. Mater. Sci..

[43] Ceperley D.M., Alder B.J. (1980). Ground state of the electron gas by a stochastic method. Phys. Rev. Lett..

[44] Perdew J., Zunger A. (1981). Self-interaction correction to density-functional approximations for many-electron systems. Phys. Rev. B.

[45] Atomistix ToolKit, version 2008.10, QuantumWise A/S. www.quantumwise.com.

[46] Büttiker M., Imry Y., Landauer R., Pinhas S. (1985). Generalized many-channel conductance formula with application to small rings. Phys. Rev. B.

[47] Stokbro K., Taylor J., Brandbyge M., Mozos J.-L., Ordejón P. (2003). Theoretical study of the nonlinear conductance of Di-thiol benzene coupled to Au(1 1 1) surfaces via thiol and thiolate bonds. Comput. Mater. Sci..

[48] Yuan S., Wang S., Mei Q., Ling Q., Wang L., Huang W. (2014). Effects of electrodes and nitrogen-atom locations on electron transport in C_59_N molecular junctions: A first-principles study. J. Phys Chem C..

