# Supplementary material for: Theoretical Studies of the Spin-Dependent Electronic Transport Properties in Ethynyl-Terminated Ferrocene Molecular Junctions
Source: Micromachines (Basel). 2018 Feb 26;9(3):95. doi: 10.3390/mi9030095 (PMC6187227; doi:10.3390/mi9030095)
Supplement: Supplementary file 1 [file micromachines-09-00095-s001.pdf]

Supplementary Materials: Theoretical Studies of the Spin-Dependent Electronic Transport Properties in Ethynyl-Terminated Ferrocene Molecular Junctions

Shundong Yuan, Shiyan Wang, Zhaoyang Kong, Zhijie Xu, Long Yang, Diansheng Wang, Qidan Ling and Yudou Wang

Energy level	Spin up	Spin down
402	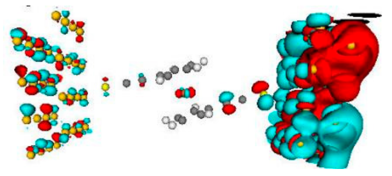	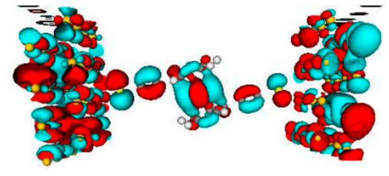
401	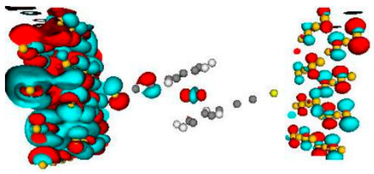	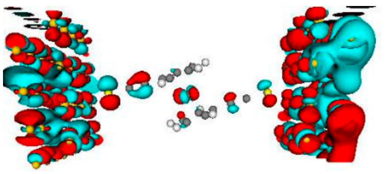
400	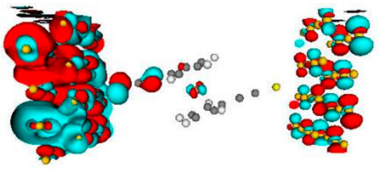	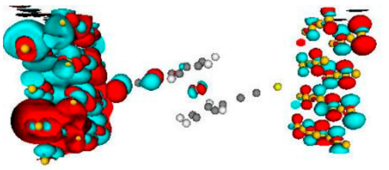
399	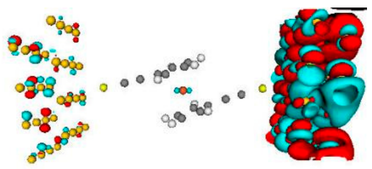	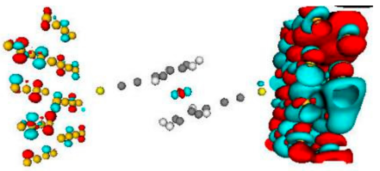
398	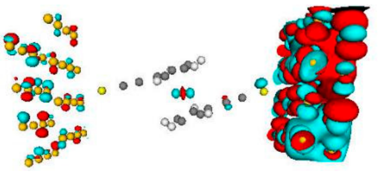	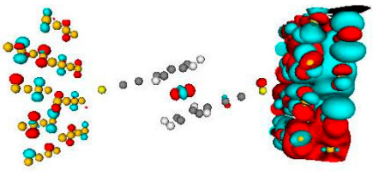
397	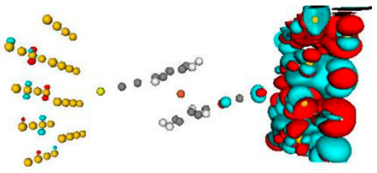	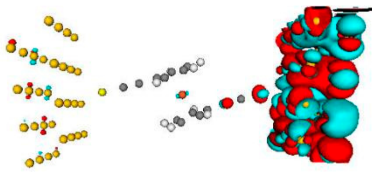
396	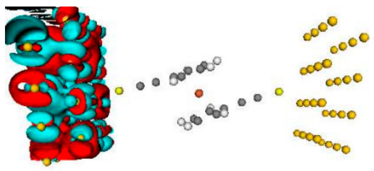	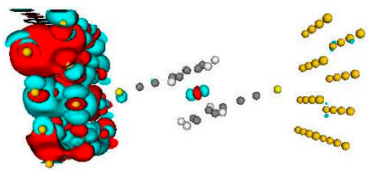

**395**	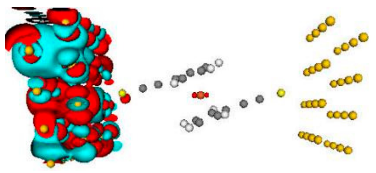	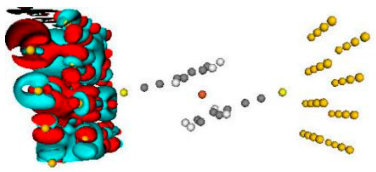
**394**	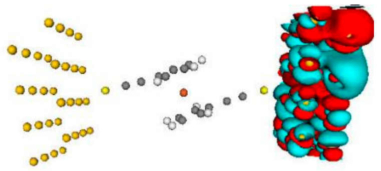	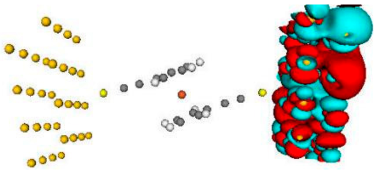
**393**	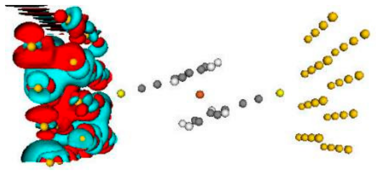	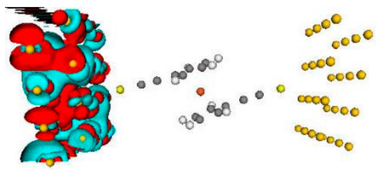
**392**	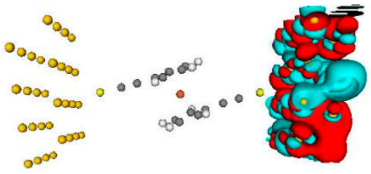	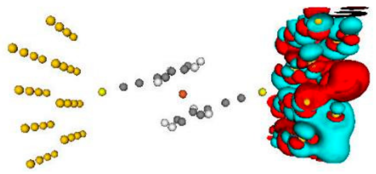
**391**	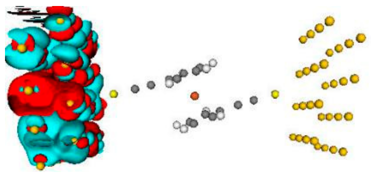	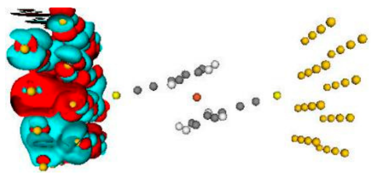

Figure S1. Spatial distribution of spin-up and spin-down MPSH states 391-402 at 0.05 V for model M2. The isovalue is 0.03.

**Energy level**	**Spin up**	**Spin down**
**406**	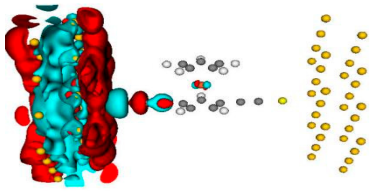	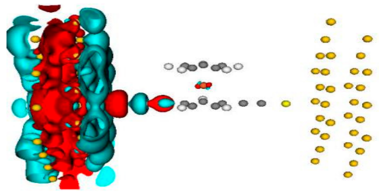
**405**	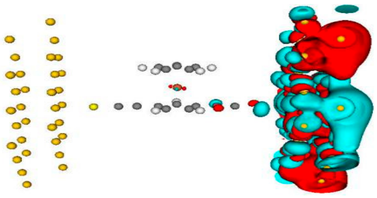	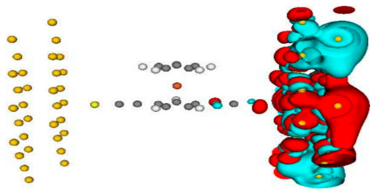
**404**	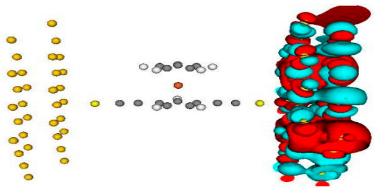	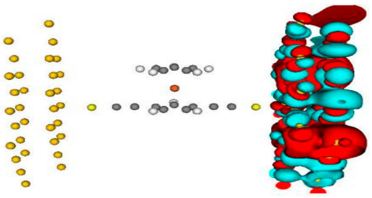
**403**	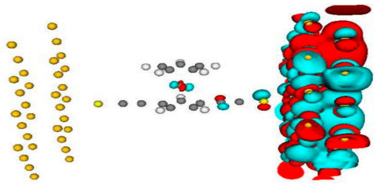	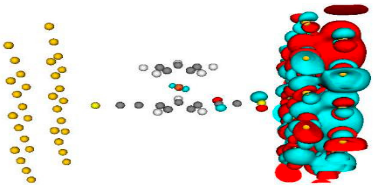
**402**	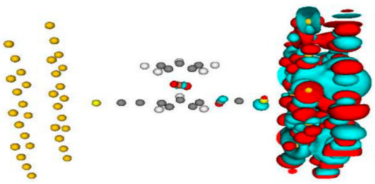	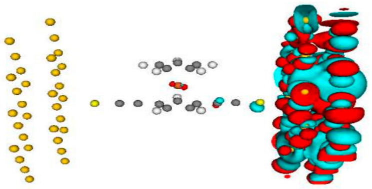
**401**	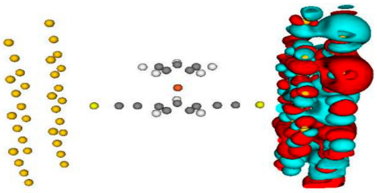	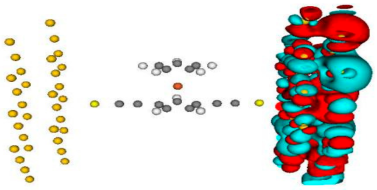
**400**	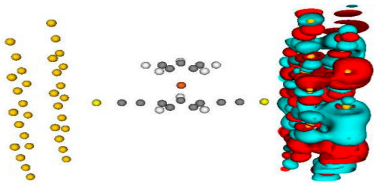	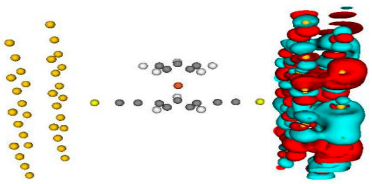
**399**	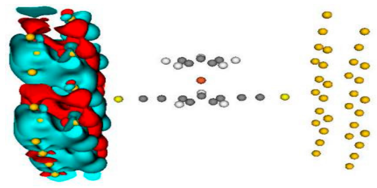	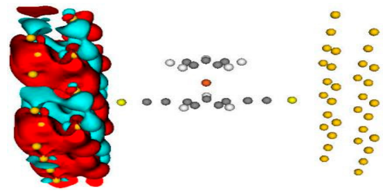

**398**	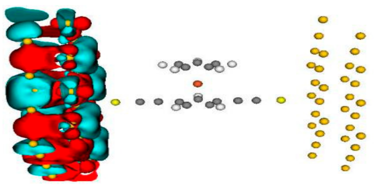	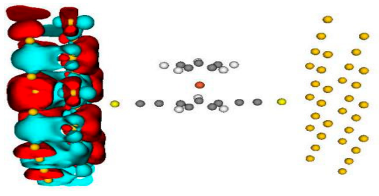
**397**	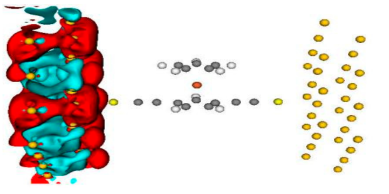	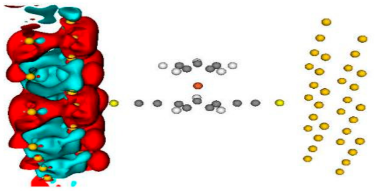
**396**	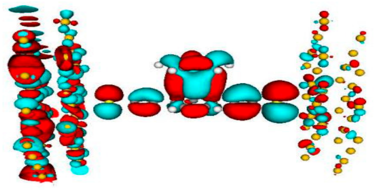	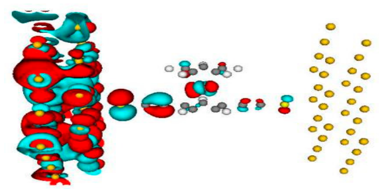
**395**	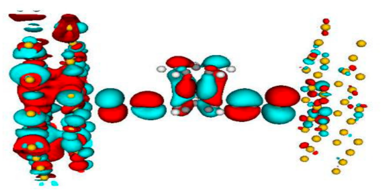	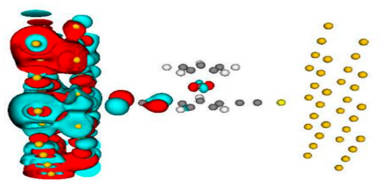
**394**	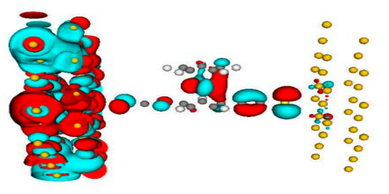	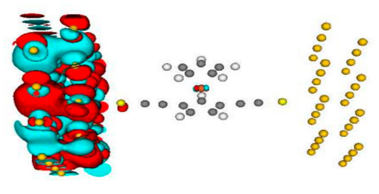
**393**	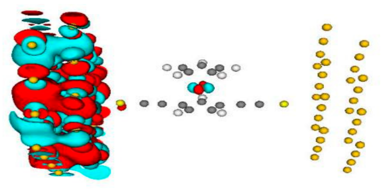	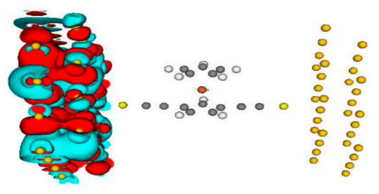
**392**	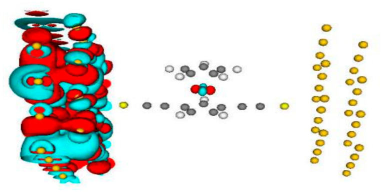	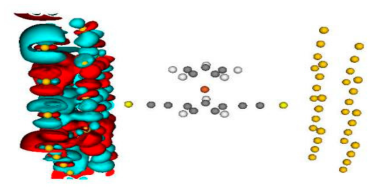
**391**	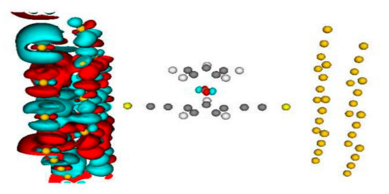	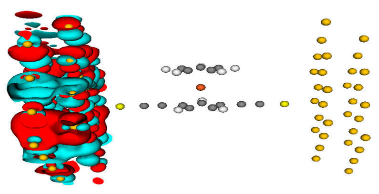

**390**	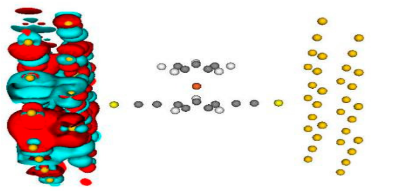	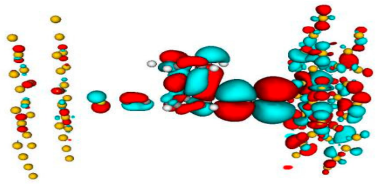

Figure S2. Spatial distribution of spin-up and spin-down MPSH states 390-406 at 0.5 V for model M1. The isovalue is 0.03.
